# The application of deep learning in abdominal trauma diagnosis by CT imaging

**DOI:** 10.1186/s13017-024-00546-7

**Published:** 2024-05-06

**Authors:** Xinru Shen, Yixin Zhou, Xueyu Shi, Shiyun Zhang, Shengwen Ding, Liangliang Ni, Xiaobing Dou, Lin Chen

**Affiliations:** 1 School of Life Sciences, Zhejiang Chinese Medical University, Hangzhou, Zhejiang PR China; 2 School of Computing and Information, Information Science, University of Pittsburgh, Pittsburgh, PA USA; 3 School of Software, Hefei University of Technology, Hefei, Anhui PR China

**Keywords:** Abdominal trauma, Computed tomography, Deep learning

## Abstract

**Background:**

Abdominal computed tomography (CT) scan is a crucial imaging modality for creating cross-sectional images of the abdominal area, particularly in cases of abdominal trauma, which is commonly encountered in traumatic injuries. However, interpreting CT images is a challenge, especially in emergency. Therefore, we developed a novel deep learning algorithm-based detection method for the initial screening of abdominal internal organ injuries.

**Methods:**

We utilized a dataset provided by the Kaggle competition, comprising 3,147 patients, of which 855 were diagnosed with abdominal trauma, accounting for 27.16% of the total patient population. Following image data pre-processing, we employed a 2D semantic segmentation model to segment the images and constructed a 2.5D classification model to assess the probability of injury for each organ. Subsequently, we evaluated the algorithm’s performance using 5k-fold cross-validation.

**Results:**

With particularly noteworthy performance in detecting renal injury on abdominal CT scans, we achieved an acceptable accuracy of 0.932 (with a positive predictive value (PPV) of 0.888, negative predictive value (NPV) of 0.943, sensitivity of 0.887, and specificity of 0.944). Furthermore, the accuracy for liver injury detection was 0.873 (with PPV of 0.789, NPV of 0.895, sensitivity of 0.789, and specificity of 0.895), while for spleen injury, it was 0.771 (with PPV of 0.630, NPV of 0.814, sensitivity of 0.626, and specificity of 0.816).

**Conclusions:**

The deep learning model demonstrated the capability to identify multiple organ injuries simultaneously on CT scans and holds potential for application in preliminary screening and adjunctive diagnosis of trauma cases beyond abdominal injuries.

## Introduction

Abdominal trauma is a serious health issue that affects the survival of the injured, especially those under age of 45 years [1], it is a common occurrence in both peaceful and hostile environments, and it mainly involves the liver, spleen, and kidney. Among all types of bodily injuries, those to the abdomen comprise from 0.4% up to 1.8% [2, 3].Despite the improvements in trauma management, abdominal trauma still poses a significant threat to the mortality of the injured, with a global variation of 1–20% [4–6]. Abdominal trauma involves a variety of injuries to the abdominal organs, which have different structures and functions. The main causes of death from abdominal trauma are massive bleeding and severe infection in the abdominal cavity. To reduce the mortality of abdominal trauma, it is essential to quickly and accurately assess the presence and extent of internal organ injury, the type and location of organ injury, and the appropriate treatment [7].The diagnosis of abdominal trauma is often challenging, as it may not be evident from physical examination, patient symptoms, or laboratory tests. The most widely adopted trauma evaluation system is the American Association for Surgery of Trauma-organ injury scale (AAST-OIS), which was first introduced in 1989, and the latest update was in 2018. It categorizes the injuries from mild to severe into grades I to V, based on the alterations in the anatomical structure of the injured organ. This scale is considered as the gold standard for trauma classification [8].

CT is indispensable for assessing suspected abdominal injuries in clinical practice, as it provides a comprehensive view of the abdomen, with a unique role in evaluating abdominal parenchymal organs, hollow organs, mesentery, omentum, and vascular injuries caused by trauma, especially when severe or multi-system injuries are suspected [9]. Based on AAST-OIS criteria, the accurate display and interpretation of CT signs are crucial for the clinical diagnosis and classification for trauma patients [10, 11]. Therefore, the standardization of CT scan images for the precise judgment of radiologists on injury signs, and the consistency of grading interpretation are essential in providing better information for therapeutic strategies.

However, explaining CT scanning on abdominal trauma typically is a complex and time-consuming process. In clinical, several factors contribute to the likelihood of errors in abdominal trauma CT diagnosis. These include imaging backlog, understaffing, visual fatigue, and overnight shifts, especially when the caseload exceeds the daily workload [12, 13]. Moreover, the complexity of the abdomen, with its multitude of organs, poses challenges for comprehensive diagnosis, especially in cases involving multiple injuries or active bleeding. Furthermore, anatomical variations and incorrect patient positioning can also lead to misdiagnosis [14]. Despite the involvement of a second radiologist in reviewing the diagnostic results, errors and missed diagnoses remain challenging in clinical practice.

To overcome this challenge, artificial intelligence (AI) technology could facilitate the prompt diagnosis of such injuries and enhance the treatment and care of patients in emergency settings. Therefore, the medical community is increasingly interested in applying AI and machine learning (ML) to assist clinicians. By deploying AI model as virtual diagnostic assistants to serve as secondary image readers, the accuracy and dependability of radiological image interpretation can be significantly enhanced. This empowers radiologists with greater confidence in their diagnostic assessments. Leveraging the feature of AI in rapidly identifying image can expedite the diagnostic process and improve clinical efficiency [15].

In this study, we developed a novel assay based on deep learning to detect severe abdominal organ damage, locate the affected organs, such as the liver, spleen, kidneys, and intestines, and identify any active intra-abdominal bleeding. The purpose of this study is to utilize AI technology to assist in the rapid diagnosis of abdominal trauma. Clinicians can utilize the high-quality image results provided by our developed deep learning model, combined with patient medical history, symptoms and signs for comprehensive and accuracy analysis. This approach facilitates faster initial screening and triage, assisting to quickly identify genuine abdominal trauma patients.

## Methods and materials

### Data acquisitions and labelling

The datasets were acquired from RSNA Abdominal Trauma Detection AI Challenge (2023) [16], which is a competition database established by the Radiological Society of North America (RSNA) for machine learning model development, in which the images are collected from 23 sites of 14 countries in six continents of more than 4,000 CT scans of patients with various types of abdominal injuries.

For the present study, we picked up 3,147 patients’ data and labelled into six sections, including three parenchymatous organs (liver, spleen, kidney), one cavity organ (intestine), and other two parts of extravasation and any injury. Each section was divided into two categories: health or injury, showed in Table 1.

**Table 1:** Classification of data

No.	Organ	status
1	Liver	Healthy
		Injury
2	Spleen	Healthy
		Injury
3	Kidney	Healthy
		Injury
4	Bowel	Healthy
		Injury
5	Extravasation	Healthy
		Injury
6	Any Injure	Healthy
		Injury

The required images were transformed from DCM (Digital Imaging and Communications in Medicine, DICOM) into Portable Network Graphics (PNG) for quantitatively simplification and visually optimization. Then, the screened images were cropped in abdominal organs on Z stacks for three-dimensional model establishment.

### Images segmentation on abdominal organs

For the rapidly segmentation and processing the liver, spleen, kidney, and intestine in CT images, we applied a 2D semantic segmentation model named U-Net. In this model we divided and extracted the organs of each CT image on every patient. Based on these results, a special semantic segmentation database for CT images was created, in which the mages were annotated on pixel-by-pixel. Then, we trained the model by EfficientNetB0 architecture based on the data set and relevant clinical knowledge, a U-Net 2D semantic segmentation model built on the EfficientNetB0 architecture was trained, and the 5kfold cross-validation was used to evaluate the model’s performance.

### Rapid diagnosis and screening of abdominal organ trauma

**Fig. 1:**
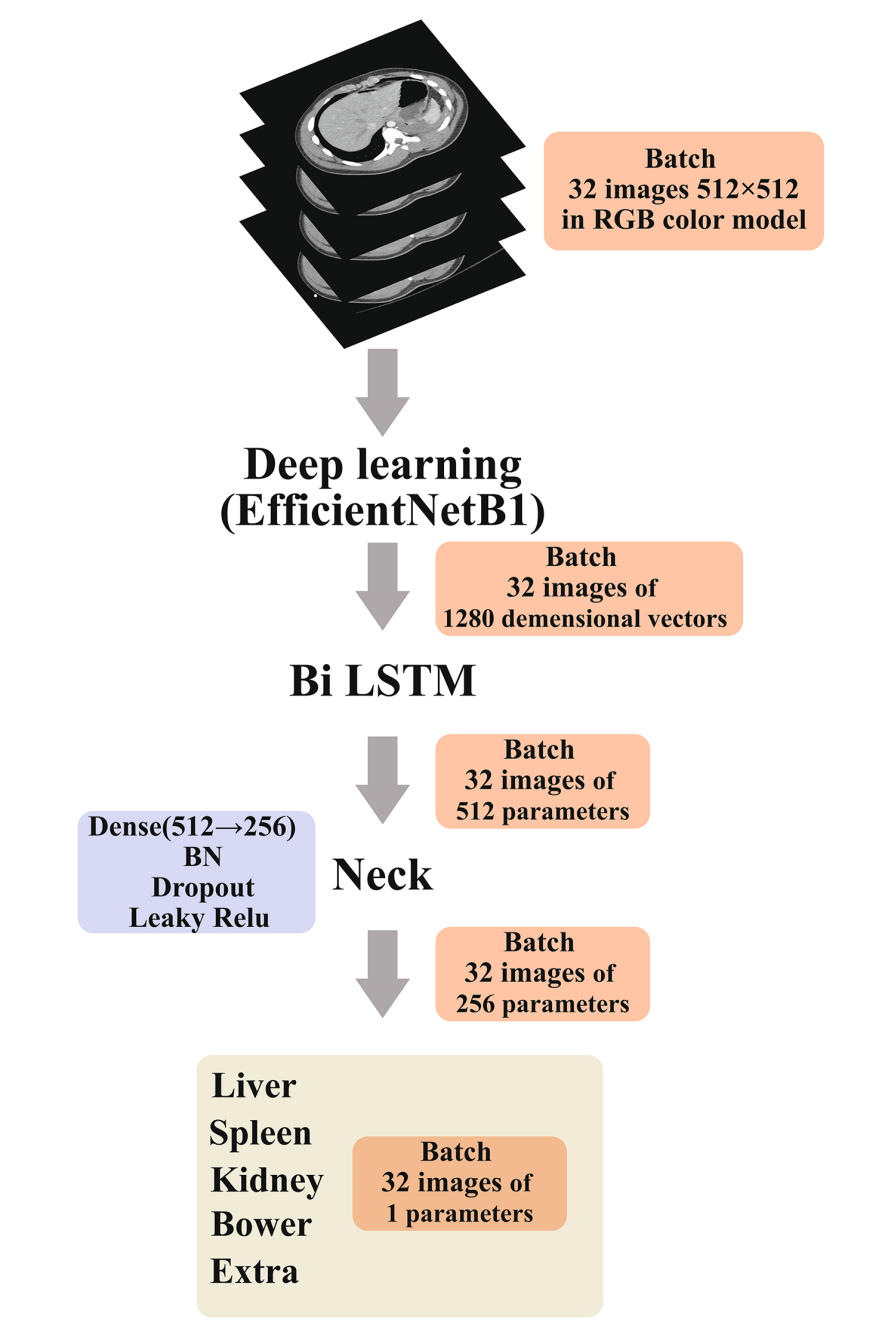
Working diagram on 2.5D model

We constructed a 2.5D semantic segmentation to enhance the analysis of spatial relationships among abdominal organs. The initial step involved identifying the commencement and termination points within each serial section where these organs were visible. We selected 32 images from every section, ensuring each organ was represented in at least four images. Images have 512 × 512 pixels in RGB color model. These images were then processed through the EfficientNetB1 model, converting them into 1280-dimensional vectors. These vectors served as inputs for a two-way Long Short-Term Memory (LSTM) Networks, facilitating the extraction of spatial dimensional features. Through the LSTM network, the feature vector of EfficientNetB1 was changed from 1280 to 512. Subsequently, we employed a Neck structure before subjecting the data to Multi-Label classification to compute the average injury probability for each organ, as showed in Fig. 1.

For evaluating how the accuracy of the algorithm model fit to the dataset, we induced the loss function, which was designed based on the weighted cross-entropy loss function for the individual organ calculation.

### Statistical analysis

We performed statistical analysis with the Python libraries NumPy, Pandas, and TensorFlow. We presented the model classification results in terms of accuracy, sensitivity, and specificity using a confusion matrix. We also assessed the model performance using the receiver operating characteristic (ROC) curve and the area under the ROC curve (AUROC). We calculated the confidence intervals of these metrics using the bootstrapping method. In addition, we use the Grad-CAM visualization algorithm to evaluate the model’s capabilities.

### Hardware and software

We developed and tested the model on a workstation running Ubuntu 18.04 that included two Intel® Xeon® Gold 6258R CPUs operating at 2.70 GHz, 768 GB of RAM, and eight NVIDIA Tesla V100 (16GB) GPUs. We used Tensorflow v2.14.0 and Python v3.9.18 to create the full process. For picture preprocessing, we used Python packages like Pydicom and OpenCV. TensorFlow, pandas, and NumPy are Python libraries that we used to perform statistical analysis. Using a confusion matrix, we displayed the model’s classification results in terms of accuracy, sensitivity, and specificity. The ROC curve and the AUROC were also used to evaluate the model’s performance. We used the bootstrapping approach to obtain these measures’ confidence intervals.

## Results

### Patients

The 3147 patients’ images were collected in the database, in which 855 (27.16%) of them had abdominal trauma. The patients who suffered from abdominal trauma were divided into the following categories: 321 patients (37.54%) had liver damage, 354 patients (41.40%) had spleen damage, 182 patients (21.29%) had kidney damage, 64 patients (7.49%), and 200 patients (23.39%) had abdominal extravasation.

### 2D semantic segmentation model worked well in images segmentation

**Fig. 2:**
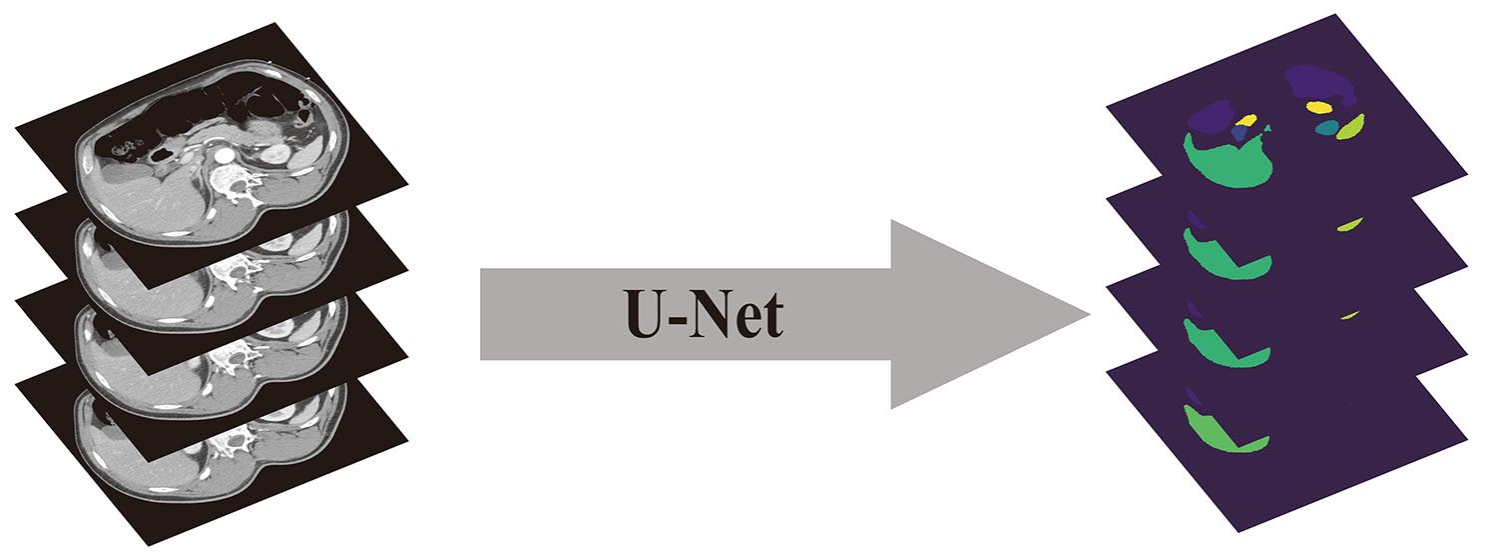
Working diagram on 2D model

The model performs well in identifying the location and category of each organ, for the location accuracy was about 85%, and the category accuracy was higher than 90%, that suggested the model can effectively accomplish the tasks of organ localization and categorization. The figure below demonstrates the organ localization using a 2D semantic segmentation model. The left image is the original CT scan, and the right image is the segmented image. The results of the validation of the 2D semantic segmentation model in the 5kfold loop are shown in the following Table 2; Fig. 2.

**Table 2:** The results of the validation of the 2D model

binary_focal_dice_loss	f1-score	iou_score	precision	recall
0.0884	0.9151	0.8516	0.9207	0.9128

### The 2.5D classification approach performs well in abdominal organs injuries diagnosis

**Fig. 3:**
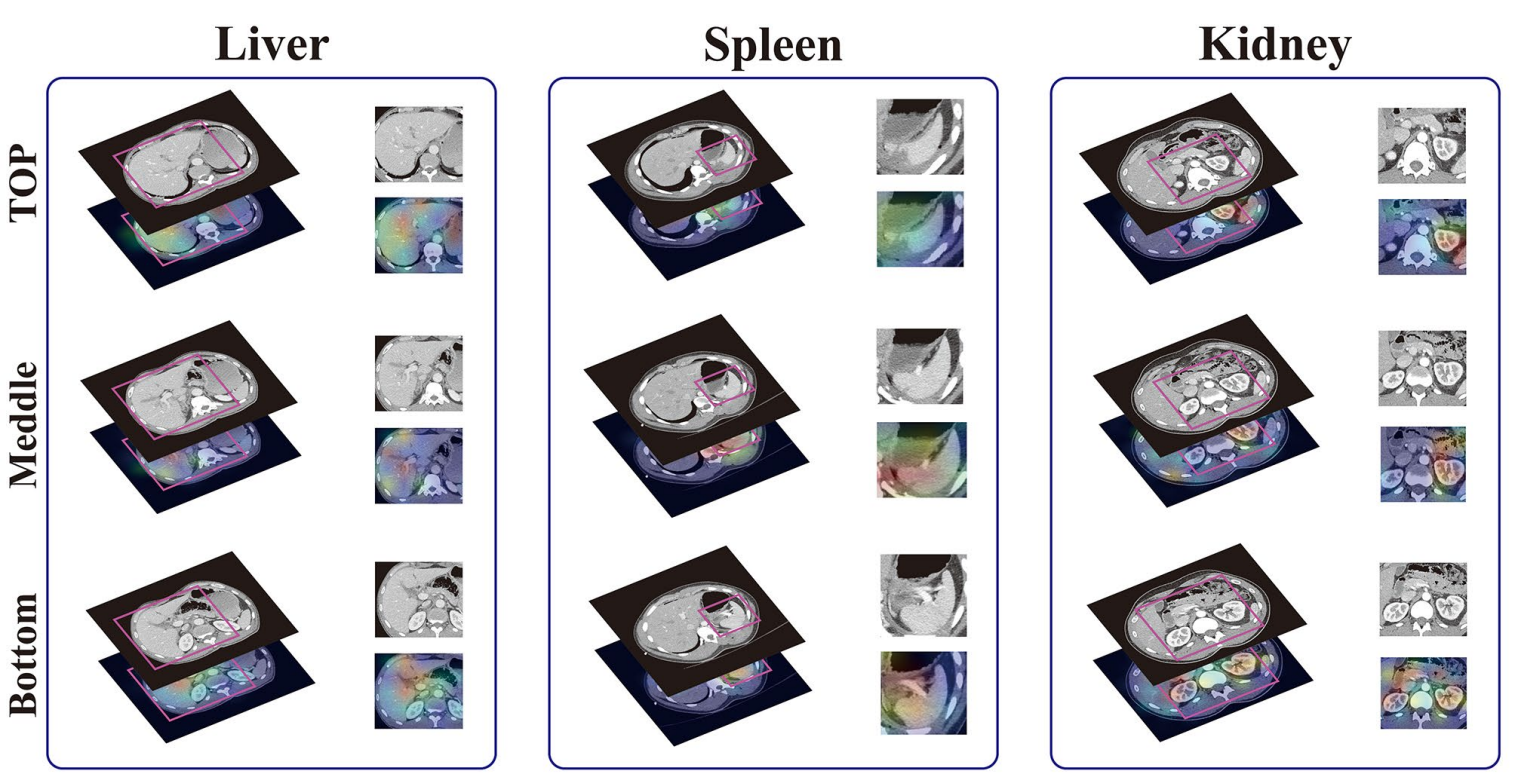
Examples of visual results of liver injury, spleen injury and kidney injury detection. The model can accurately locate the damaged parts of liver, spleen and kidney

The 2.5D classification model assessed the injury probability for five organs: liver, spleen, kidney, intestine, and exosmosis. Additionally, an “any injury” category was included, representing the maximum probabilities across the five organs. Seven indexes were employed for model classification: area under the curve (AUC), accuracy, PPV, NPV, sensitivity, and specificity. Notably, specificity indicates the accuracy of identifying healthy organs when the sensitivity for detecting diseased organs is set at 90% in the 2.5D model. To evaluate the model’s capabilities, we applied the Grad-CAM visualization algorithm to the test set. The resulting visual heat maps demonstrated the model’s ability to detect organ damage. Figure 3 illustrates examples of the visualized results focusing on the liver, spleen, and kidney.

#### Liver diagnosis

**Fig. 4:**
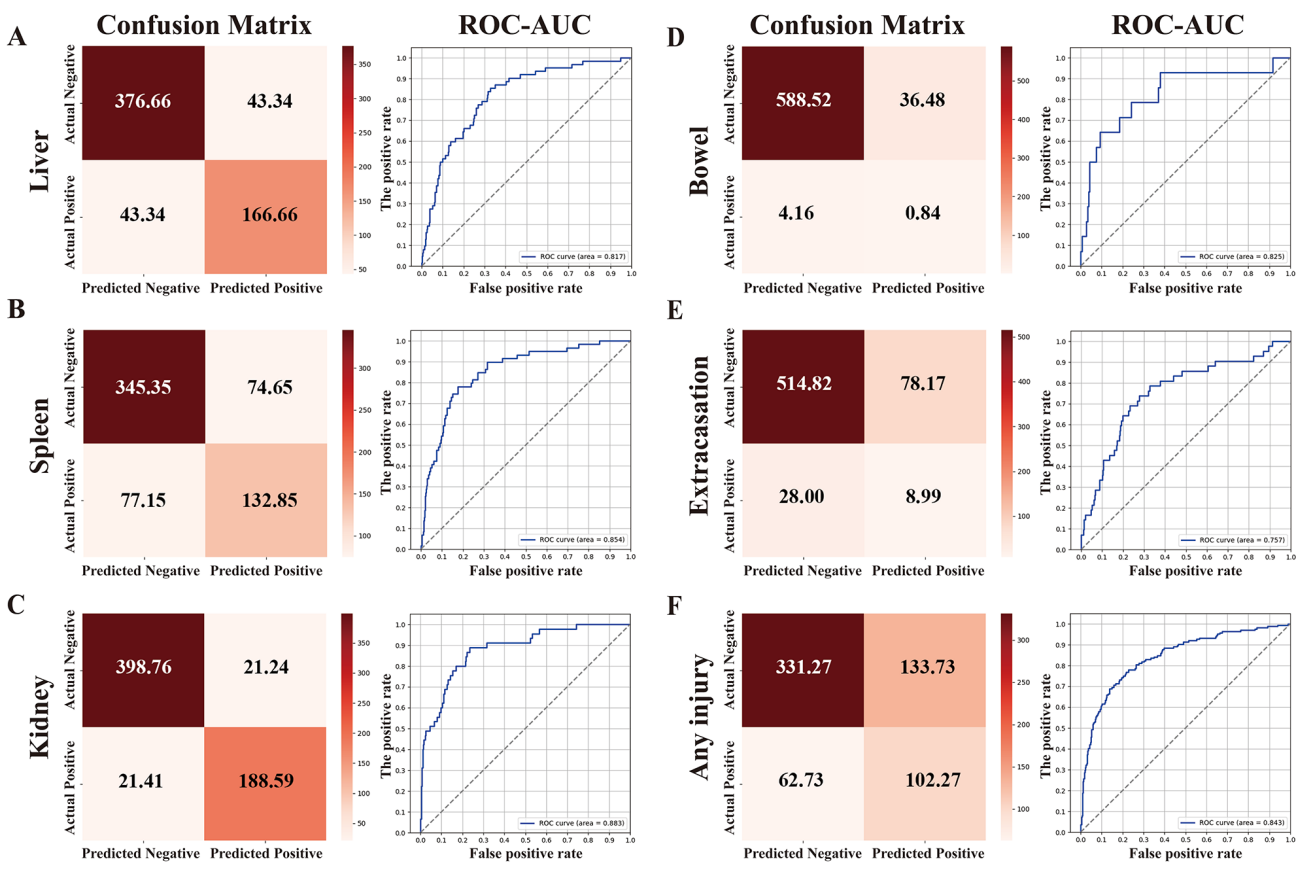
The results of confusion matrix and ROC-AUC.

The 2.5D classification model achieved high accuracy and sensitivity in diagnosing liver injury on CT scans. The specific results are shown in Table 3; Fig. 4A. The AUC and ACC were 0.817 (0.763–0.868) and 0.873 (0.848–0.898), respectively. The PPV and NPV were 0.789 (0.77–0.809) and 0.895 (0.885–0.904), respectively. The sensitivity and specificity were 0.789 (0.77–0.809) and 0.895 (0.885–0.904), respectively. These results show that the model can effectively detect liver injury and facilitate clinical decision-making.

**Table 3:** The results of diagnosis

No.	Metrics/ Organs	Liver	Spleen	Kidney	Bowel	Extravasation	Any Injury
**1**	**AUC**	0.817	0.848	0.882	0.83	0.757	0.843
	**95% CI**	0.763–0.868	0.795–0.895	0.823–0.929	0.699–0.942	0.67–0.833	0.803–0.88
**2**	**ACC**	0.873	0.771	0.932	0.978	0.935	0.795
	**95% CI**	0.848–0.898	0.74–0.803	0.911–0.951	0.965–0.989	0.916–0.952	0.762–0.827
**3**	**PPV**	0.789	0.63	0.888	0.056	0.114	0.438
	**95% CI**	0.77–0.809	0.61–0.65	0.87–0.904	0.027–0.091	0.082–0.149	0.387–0.488
**4**	**NPV**	0.895	0.814	0.943	0.98	0.941	0.852
	**95% CI**	0.885–0.904	0.804–0.823	0.935–0.952	0.969–0.99	0.925–0.958	0.825–0.878
**5**	**Sensitivity**	0.789	0.626	0.887	0.149	0.247	0.653
	**95% CI**	0.77–0.809	0.606–0.645	0.87–0.904	0.104–0.203	0.209–0.29	0.611–0.696
**6**	**Specificity**	0.895	0.816	0.944	0.943	0.863	0.705
	**95% CI**	0.885–0.904	0.807–0.826	0.935–0.952	0.939–0.947	0.855–0.87	0.685–0.724

#### Spleen diagnosis

The 2.5D classification model showed moderate accuracy and sensitivity in detecting spleen injury on CT scans. The specific results are shown in Table 3; Fig. 4B. The AUC and ACC were 0.848 (0.795–0.895) and 0.771 (0.74–0.803), respectively. The PPV and NPV were 0.63 (0.61–0.65) and 0.814 (0.804–0.823), respectively. The sensitivity and specificity were 0.626 (0.606–0.645) and 0.816 (0.807–0.826), respectively. These results indicate that the model can reliably exclude spleen injury and save medical resources. However, the model has low PPV and sensitivity, and its performance in identifying positive cases can be improved.

#### Renal diagnosis

The 2.5D classification model achieved high performance in detecting kidney injury on CT scans. The specific results are shown in Table 3; Fig. 4C. The AUC and ACC were 0.882 (0.823–0.929) and 0.932 (0.911–0.951), respectively. The PPV and NPV were 0.888 (0.87–0.904) and 0.943 (0.935–0.952), respectively. The sensitivity and specificity were 0.887 (0.87–0.904) and 0.944 (0.935–0.952), respectively. These results indicate that the model can effectively diagnose kidney injury and support clinical decision-making.

#### Intestinal diagnosis

The 2.5D classification model showed high NPV and specificity in excluding intestinal injury on CT scans. The specific results are shown in Table 3; Fig. 4D. The AUC and ACC were 0.83 (0.699–0.942) and 0.978 (0.965–0.989), respectively. The PPV and NPV were 0.056 (0.027–0.091) and 0.98 (0.969–0.99), respectively. The sensitivity and specificity were 0.149 (0.104–0.203) and 0.943 (0.939–0.947), respectively. These results suggest that the model can reliably rule out intestinal injury, but has low PPV and sensitivity in detecting positive cases.

#### Extravasation diagnosis

The 2.5D classification model showed high NPV and specificity in excluding extravasation on CT scans. The specific results are shown in Table 3; Fig. 4E. The AUC and ACC were 0.757 (0.67–0.833) and 0.935 (0.916–0.952), respectively. The PPV and NPV were 0.114 (0.082–0.149) and 0.941 (0.925–0.958), respectively. The sensitivity and specificity were 0.247 (0.209–0.29) and 0.863 (0.855–0.87), respectively. These results suggest that the model can reliably rule out extravasation, but the low PPV and sensitivity in detecting positive cases. The low AUC and high ACC indicate that the data set of extravasation was imbalanced, with a large proportion of negative cases, leading to the model misclassifying some positive cases as negative.

#### Arbitrary damage diagnosis

The 2.5D classification model showed high NPV and specificity in excluding any injury on CT scans. The specific results are shown in Table 3; Fig. 4F. The AUC and ACC were 0.843 (0.803–0.88) and 0.795 (0.762–0.827), respectively. The PPV and NPV were 0.438 (0.387–0.488) and 0.852 (0.825–0.878), respectively. The sensitivity and specificity were 0.653 (0.611–0.696) and 0.705 (0.685–0.724), respectively. These results suggest that the model can reliably rule out any injury, but has low PPV and sensitivity in detecting positive cases. We hypothesized that the low PPV and sensitivity were related to the low performance of the model in diagnosing intestinal and extravasation injuries.

## Discussion

In this study, we developed an algorithm that can detect injuries in five abdominal organs: liver, spleen, kidney, intestine, and extravasation. Our results demonstrate that the algorithm can accurately diagnose parenchymal organ injuries. The algorithm can localize the abdominal organs and then detect the injuries in each organ simultaneously, which can assist clinicians in efficient screening and triage, facilitate the treatment of trauma patients, and avoid the waste of medical resources.

Furthermore, the algorithm performed best in identifying kidney injury on abdominal CT scans, with an ACC of 0.932 (PPV: 0.888; NPV: 0.943; Sensitivity: 0.887; Specificity: 0.944). It also showed good performance in diagnosing liver and spleen injuries, with an ACC of 0.873 (PPV: 0.789; NPV: 0.895; Sensitivity: 0.789; Specificity: 0.895) and 0.771 (PPV: 0.63; NPV: 0.814; Sensitivity: 0.626; Specificity: 0.816), respectively.

In clinical practice, radiologists’ diagnostic focus and efficiency can be impacted by various factors, including fatigue and time pressure. Studies have shown that the error rates of radiologists in abdominal CT diagnosis fluctuate throughout the day and week. Specifically, during the workweek, error rates are highest later in the morning and at the end of the workday, with Mondays showing higher rates compared to other days [17]. Additionally, there are notable variations in expertise and experience among radiologists of different ages and qualifications. Studies have found that less experienced radiologists may have error rates as high as 32% in diagnosing abdominal solid organ CT images under busy conditions [18–20]. In contrast, diagnostic models based on deep learning exhibit robust stability and rapid speed, unaffected by subjective or objective factors, which can operate continuously for 24 h a day. During our study, we uploaded 3,147 patients’ CT images for the model learning, which cost about 5 h until the diagnosis completion. For individual patient, it takes seconds or minutes to finish the analysis, the time depends on the difference and quality of each CT image.

Previous studies have applied deep learning algorithms to diagnose specific abdominal injuries, such as kidney segmentation [21], splenic laceration [22], liver laceration [23, 24], and abdominal hemorrhage [25]. However, none of these studies have attempted to detect multiple organ injuries in trauma patients. Therefore, we developed a deep learning algorithm that can detect injuries in five different abdominal organs at once. We used a 2D semantic segmentation model to extract the organs from the CT images, and then a 2.5D classification model to predict the injury probability of each organ. This approach improved the speed and accuracy of the algorithm.

We developed an algorithm that requires large amounts of accurately labeled data to achieve high performance, to facilitate the labeling process for the clinicians and enhance the results of the automatic detection algorithm. The dataset for CT examinations in this study included both conventional and enhanced CT scans. We conducted a normalization on the data to avoid the effect of the contrast agent usage in patient before uploaded to the model. A 2.5D classification model had been introduced instead of a 3D model, which could recognize the small data sets, for reduction parameters numbers and prevention the overfitting while preserving the model performance. The LSTM was included as well, for the components could performed spatial analysis on the input serial CT images and capture the spatial relationships among the images.

To improve the diversity and quality of the data set and address the data imbalance issue, we applied various data augmentation techniques that mimic different scenarios that can affect the quality of CT images and help the model cope with reality variations. With the basic geometric transformations, such as horizontal and vertical flipping, which helped to distinguish the same anatomical structures in different orientation caused by scanning angles.

The Geometric transformation technology, blur technology and random Gaussian noise was applied for data enhancement, which can effectively enrich the training data set, resist the occurrence of over-fitting, and enable the model to make correct fitting when facing new images, instead of blindly limiting it to some known images.

Over all, deep learning has been widely applied to clinical data analysis, especially in image processing [26]. It has advanced the field of medical imaging by enabling the identification, classification, and quantification of patterns in various modalities [27], and quantitative assessment of blunt liver trauma in children [28], which can provide clinicians with accurate and fast diagnostic assistant [29]. Our deep learning model provided a high-quality image analysis result that helps clinicians perform quick screening and triage, identify patients with abdominal trauma, to improve medical efficiency and save medical resources when in natural disasters or mass accidents. Moreover, the model has the potential to be applied to the CT diagnosis of other diseases.

## Conclusions

This deep learning model can be used to identify multiple organ injuries simultaneously on CT, and may be further applied to the preliminary screening and auxiliary diagnosis of other trauma scenes. While our model has shown promising results, it still has limitations. One of the main areas for improvement is the need for a larger dataset of CT images for more robust algorithm training. Additionally, we aim to explore methods for optimizing the algorithm to enhance the predictive ability of the model. Moving forward, our focus will be on continually augmenting the dataset by incorporating more CT images to further enhance the accuracy and speed of the model.

## Data Availability

No datasets were generated or analysed during the current study.

## Abbreviations

CT: Computed tomography
PPV: Positive predictive value
NPV: Negative predictive value
AAST-OIS: American association for surgery of trauma-organ injury scale
AI: Artificial intelligence
ML: Machine learning
RSNA: Radiological society of North America
DICOM: Digital imaging and communications in medicine
PNG: Portable network graphics
LSTM: Long short-term memory
ROC: Receiver operating characteristic
AUROC: Area under the roc curve
